# Analysis of Suppressor and Non-Suppressor FOXP3+ T Cells in HIV-1-Infected Patients

**DOI:** 10.1371/journal.pone.0052580

**Published:** 2012-12-20

**Authors:** Lourdes Arruvito, Juan Sabatté, Julieta Pandolfi, Plácida Baz, Luis A. Billordo, Maria B. Lasala, Horacio Salomón, Jorge Geffner, Leonardo Fainboim

**Affiliations:** 1 Institute of Immunology, Genetics and Metabolism (INIGEM), Clinical Hospital, University of Buenos Aires, National Council for Scientific and Technological Research, Buenos Aires, Argentina; 2 Institute for Biomedical Research on Retroviruses and AIDS, Department of Microbiology, School of Medicine, University of Buenos Aires, Buenos Aires, Argentina; 3 Department of Infectious Diseases, Clinical Hospital, University of Buenos Aires, Buenos Aires, Argentina; New York University, United States of America

## Abstract

Recently, it was shown that peripheral blood FOXP3+CD4+ T cells are composed of three phenotypic and functionally distinct subpopulations. Two of them having in vitro suppressive effects were characterized as resting Treg cells (rTregs) and activated Treg cells (aTregs). A third subset, identified as FOXP3+ non-Tregs, does not display any suppressor activity and produce high levels of Th1 and Th17 cytokines upon stimulation. In the present study we focus on the characteristics of these three subsets of FOXP3+CD4+ T cells in untreated HIV-1-infected patients. We found that the absolute counts of rTregs, aTregs and FOXP3+ non-Tregs were reduced in HIV-1 patients compared with healthy donors. The relative frequency of rTregs and aTregs was similar in HIV-1 patients and healthy donors, while the frequency of FOXP3+ non-Tregs was significantly higher in HIV-1 patients, reaching a maximum in those patients with the lower values of CD4 counts. Contrasting with the observations made in FOXP3- CD4+ T cells, we did not find a negative correlation between the number of rTregs, aTregs or FOXP3+ non-Tregs and virus load. Studies performed with either whole PBMCs or sorted aTregs and FOXP3+ non-Tregs cells showed that these two populations of FOXP3+ T cells were highly permissive to HIV-1 infection. Upon infection, FOXP3+ non-Tregs markedly down-regulates its capacity to produce Th1 and Th17 cytokines, however, they retain the ability to produce substantial amounts of Th2 cytokines. This suggests that FOXP3+ non-Tregs might contribute to the polarization of CD4+ T cells into a Th2 profile, predictive of a poor outcome of HIV-1-infected patients.

## Introduction

Regulatory T cells (Tregs) have been characterized as CD4+ T cells expressing CD25 and FOXP3 and very low amounts of CD127, which excludes naive and memory conventional T cells [1], [2], [3], [4]. It was recently reported that FOXP3+CD4+ T cells include three phenotypic and functionally distinct cellular subpopulations; two of them having in vitro suppressive activity were characterized as resting Treg cells (rTregs) or FOXP3^low^CD45RA+ cells and activated Tregs (aTregs) or FOXP3^high^CD45RA- cells. A third subset of FOXP3^low^CD45RA- cells was found to be a cytokine-secreting cell population without suppressor activity, and was identified as FOXP3+ non-Tregs [5].

HIV-1 infection is associated with a progressive loss of CD4+ T cells and immune hyperactivation [6], [7]. FOXP3+ Tregs are able to control excessive immune activation, limiting tissue damage, and suppressing antigen-specific immune responses against pathogens [8], [9]. A large number of reports have analyzed the presence and function of Tregs in HIV-1-infected patients [10], [11], [12], [13], [14], [15], [16]. However, these reports have assumed that all FOXP3+CD4+ T cells display a suppressor phenotype which leads to a misunderstanding about the role of regulatory T cells in the pathogenesis of HIV-1 infection. The present study was designed to examine the different behaviors of FOXP3+ Tregs, and FOXP3+ non-Tregs in HIV-1-infected patients.

## Materials and Methods

### Study Participants

The study included 55 adult untreated HIV-1-infected patients and 27 adult uninfected individuals. HIV-1-infected patients were recruited from the AIDS National Center and from the Division of Infectious Diseases, Clinical Hospital, School of Medicine, Buenos Aires University, after giving written informed consent. Characteristics of the patient cohort are shown in Table 1. Ethical approval for this study was from the Institutional Ethics Committee (Clinical Hospital, School of Medicine, Buenos Aires) in accordance with the Declaration of Helsinki. All patients were negative for serological markers of concomitant chronic hepatitis B or C infection. To avoid Treg cells variation during the menstrual cycle [17], we only recruited age-matched male patients and controls. Blood samples were collected in EDTA tubes, and PBMCs were isolated through a Ficoll-Hypaque (Amersham) density gradient centrifugation. Quantitative determination of leukocytes was performed in a Coulter STKS hematologic analyzer (Diamond Diagnostics). Plasma viral loads (VL) were measured by the HIV-1 Amplicor Monitor Ultra sensitive method (Roche) with a lower limit of detection of 50 RNA copies/mL.

**Table 1:** Characteristics of the cohort of healthy donors and HIV-1-infected patients included in the study.

	HD	HIV-1		
		All HIV-1	High CD4	Low CD4
	*n 27*	*n 55*	*n 35*	*n 20*
**Age (years)** #	38 (21–60)	35 (21–65)	38 (21–65)	36 (21–60)
**CD4 count (cell/µl)** #	965 (951–987)	404 (273–530)***	478 (426–573)***	239 (135–297)***
**Viral Load (Log_10_ cp/mL)** #	N/A	4.4 (4.1–4.7)	4.2 (3.9–4.7)	4.6 (4.4–4.8)**

# Data represents the median (IQR). Only male healthy donors and HIV-1-infected patients were included in this study.

** p<0.01,

*** p<0.0001, N/A = not applicable. *P* values were determined by comparison with healthy donors (except VL that shows differences between the High and Low group). The Mann-Whitney test was used to compare two groups and Kruskal-Wallis test followed by Dunn multiple comparison post test was used to compare three groups.

### Cell Sorting

CD4+ T cells were purified by negative selection by using CD4+ T cell MACS beads (Miltenyi Biotec), following manufacturer’s instructions. The different subsets of FOXP3+ T cells were isolated as live cells as previously described [5] by staining purified CD4+ T cells with anti-CD4 PerCP, anti-CD25 PE and anti-CD45RA FITC antibodies (all from BD Biosciences) and sorted with a FACSAria II flow cytometer (Becton Dickinson), yielding five populations: CD25+CD45RA+ (rTregs), CD25^high^CD45RA- (aTregs), CD25^low^CD45RA- (FOXP3+ non-Tregs), CD25-CD45RA+ (naive) and CD25-CD45RA- (memory). Cells were collected into RPMI 1640 medium (Hyclone) plus 50% heat-inactivated fetal calf serum and washed once for further studies. The purity of each sorted population was higher than 98% in all the experiments. After isolation, the expression of FOXP3 in sorted cells was analyzed in each population by flow cytometry. FOXP3 was detected in >90% of aTregs and >80% of either rTregs or FOXP3+ non-Tregs. By contrast, only marginal expression of FOXP3 (<0.5%) was detected in conventional naive and memory T cells.

### FACS Analysis

Freshly isolated or in vitro-cultured cells were stained with anti-CD4 (-PerCP or -APC), anti-CD25 (-PE or -FITC), anti-CD45RA (-PE-Cy7 or -FITC), anti-CD127 (-PE), anti-CD195 (anti-CCR5-APC), anti-CD184 (anti-CXCR4-PE), anti-Ki-67 (-FITC), or anti-FOXP3 (-PE or -Alexa Fluor 488) antibodies (all from BD Biosciences). Data were acquired using a FACSAria II (Becton Dickinson) and was analyzed with FlowJo software.

### Suppression Assay

The suppressive capacity of each fraction of cells purified by cell sorting was assayed as described [17]. In brief, 3×10^4^ purified naive T cells were cultured with autologous APCs (3×10^4^ cells, obtained from CD3-depleted PBMCs; Dynal Biotech) and 3×10^4^ cells from each sorted fraction: autologous rTregs, aTregs and FOXP3+ non-Tregs. Cells were stimulated with mytomicin-treated allogeneic PBMCs (3×10^4^), and cultured in a 96-well round-bottom plate for 5 days. Cell proliferation was measured by [^3^H] thymydine uptake (Perkin ElmerLife).

### HIV-1 Stocks

Viruses were obtained from the AIDS Research and Reference Reagent Program (NIH). CCR5-using HIV-1 BaL was grown on IL-2 (10 U/ml; R&D Systems) plus PHA (10 µg/ml)-stimulated PBMCs. CXCR4-using HIV-1 IIIB was obtained from H9HTLV-IIIB supernatants. The viruses were concentrated by ultracentrifugation at 28,000 rpm for 90 min at 4°C (Beckman Instruments) and the virus pellet was suspended in RPMI 1640 medium. Levels of p24 antigen were determined by ELISA (Biomerieux), and virus input into assays was a function of p24 antigen concentration.

### Infection Assays

Whole PBMCs or the five populations of CD4+ T cells purified by cell sorting as described above (rTregs, aTregs, FOXP3+ non-Tregs, naive and memory T cells), were activated with PHA (10 µg/ml) for 2 days and then infected with HIV-1 BaL or HIV-1 IIIB at different MOI during 90 min at 37°C. Cells were washed twice and cultured for 9 days in 96-well flat-bottom plates in 200 µl of culture medium supplemented with IL-2 (20 ng/ml). Supernatants and cells were collected at different time points post-infection. The quantification of p24 antigen in the supernatants was performed by ELISA (sensitivity limit of 5,9 pg/ml; Biomerieux). Intracellular p24 antigen was evaluated by flow cytometry using the KC57-FITC antibody (Beckman-Coulter). Uninfected cells were used as controls. Data acquired using a FACSAria II were analyzed with FlowJo software.

### Quantification of Cytokines in Cell Supernatants

The five different subsets of CD4+ T cells were sorted and stimulated for 5 h with PMA (50 ng/ml)/ionomycin (1 µg/ml). The concentrations of cytokines in cell-free supernatants were quantified according to the manufacturer’s instructions using the Bio-PlexTM 200 system (Bio-Rad). In some experiments sorted FOXP3+ non-Tregs were activated by PHA (10 µg/ml) for 2 days. Then, cells were infected by HIV-1 and cultured for 9 days. After this period, cells were re-stimulated by PMA/ionomycin, and the production of cytokines was analyzed in cell supernatants after 5 h of incubation.

### Statistical Analysis

Statistical analyses were performed using GraphPad Prism software. Two groups were compared using the non-parametric Mann-Whitney test and three or more groups were compared using the Kruskall-Wallis test followed by Dunn multiple comparison test. Spearman’s test was used to analyze correlations between the absolute number of cells and CD4 count or VL. A p value <0.05 was considered statistically significant.

## Results and Discussion

### Functional Analysis of the Different Populations of FOXP3+CD4+ T Cells in Healthy Donors

**Figure 1:**
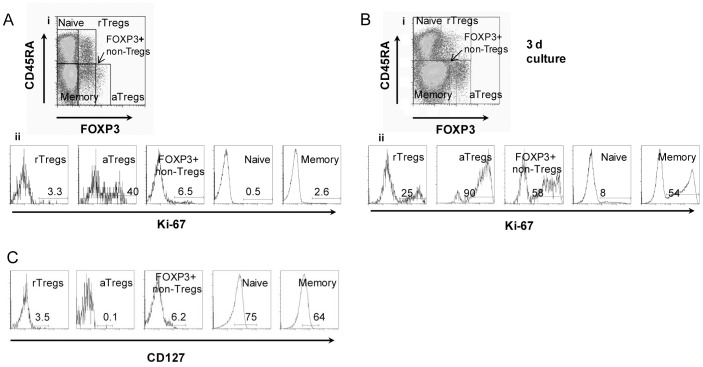
Identification of rTregs, aTregs, and FOXP3+ non-Tregs in healthy donors by flow cytometry. Ai. Based on the expression of CD45RA and FOXP3, five subsets of CD4+T cells are defined: FOXP3^low^CD45RA+ (rTregs), FOXP3^high^CD45RA- (aTregs), FOXP3^low^CD45RA- (FOXP3+ non-Tregs), FOXP3-CD45RA+ (naive T cells) and FOXP3-CD45RA- (memory T cells). Representative dot plots of CD45RA and FOXP3 expression on CD4+ T cells performed on PBMCs (n = 27) **Aii.** Representative histograms showing the expression of the proliferation marker Ki-67 for each cellular population defined in Ai. **Bi.** Representative dot plot of CD45RA and FOXP3 expression on CD4+ T cells performed on PBMCs activated during three days by using anti-CD3/CD28 antibodies. **Bii**. Representative histograms showing the expression of the proliferation marker Ki-67 for each cell population, after activation during three days with anti-CD3/CD28 antibodies. **C.** Histograms showing the expression of CD127 for each cellular subset. Aii, Bi, Bii, and C: data are representative of six independent experiments.

The different subsets of FOXP3+CD4+ T cells (rTregs, aTregs, and FOXP3+ non-Tregs) and FOXP3-CD4+ T cells (naive and memory), were analyzed following staining of PBMCs with the combination of anti-CD4, anti-CD45RA and anti-FOXP3 antibodies proposed by Miyara et al. [5]. Representative dot plots showing the three populations of FOXP3+CD4+ T cells are shown in Fig. 1Ai. The proliferative status of each subpopulation was then assessed by measuring the expression of Ki-67, which is expressed at higher levels in proliferating cells. In agreement with the observations reported by Miyara and coworkers [5] we found that at the steady state Ki-67 was detected in almost half of aTregs, but hardly detected in rTregs and FOXP3+ non-Tregs (Fig. 1Aii), while after 72 h-stimulation with anti-CD3/CD28 antibodies most aTregs and almost half of the FOXP3+ non-Tregs were actively proliferating. A low proliferative response was still detected in rTregs (Fig. 1B). As expected, the different subsets of FOXP3+ Tregs and FOXP3+ non-Tregs, but not naive or memory T cells, were mostly negative for the expression of CD127 (α chain of the interleukin 7 receptor) (Fig. 1C). From these experiments, we conclude that the three populations of FOXP3+CD4+ T cells are not anergic and can proliferate in response to activating stimuli.

**Figure 2:**
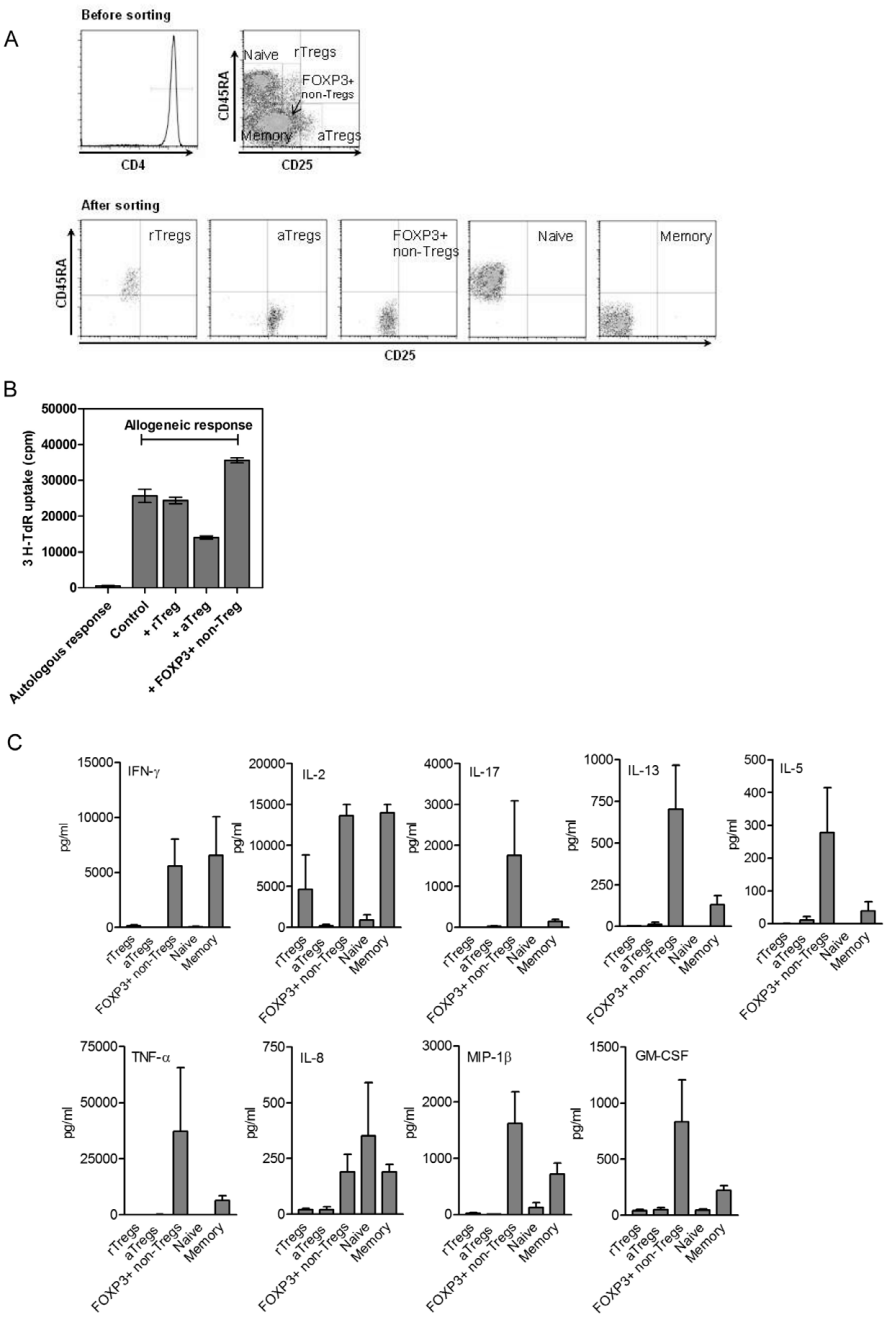
Functional characterization of FOXP3+ T cells in healthy donors. **A.** Cell sorting strategy to isolate rTregs, aTregs, FOXP3+ non-Tregs, naive and memory T cells as live cells. **Top Panel.** A representative histogram showing purified CD4+ T cells isolated from a healthy donor by negative selection. Cells were stained before sorting with anti-CD4, anti-CD25 and anti-CD45RA antibodies. rTreg cells were identified as CD25^low^CD45RA+, aTreg cells as CD25^high^CD45RA-, FOXP3+ non-Tregs as CD25^low^CD45RA-, naive T cells as CD25-CD45RA+ and memory T cells as CD25-CD45RA-. **Bottom panel.** Dot plots of each cellular subset after sorting. The purity of the sorted populations was >98% in all the experiments. The expression of FOXP3 in sorted cells was detected in >90% of aTregs and >80% of either rTregs or FOXP3+ non-Tregs. Only a marginal expression of FOXP3 (<0.5%) was observed in conventional naive and memory T cells. **B.** The ability of each population of FOXP3+ T cells to suppress the mixed lymphocyte reaction was assessed as described in Materials and Methods. Autologous response or allogeneic responses without the addition of isolated FOXP3+ T cells are included as controls. A representative experiment (n = 3) is shown. **C.** Determination of the levels of cytokines and chemokines secreted by each activated T cell subset by the Bioplex system. rTregs, aTregs, FOXP3+ non-Tregs, naive and memory T cells were purified from PBMCs by cell sorting. Then, they were stimulated by PMA/ionomycin for 5 h, and the supernatants were harvested. The results are expressed as the mean ± SEM of six independent experiments.

It has been shown [5] that the three populations of peripheral blood FOXP3+CD4+ T cells can be distinctly separated by the combination of CD25 and CD45RA staining. Thus, to isolate these different cell subsets as live cells, CD4+ T cells were sorted as CD25^low^CD45RA+ cells (rTregs), CD25^high^CD45RA- cells (aTregs), and CD25^low^CD45RA- cells (FOXP3+ non-Tregs). The cell sorting strategy is summarized in Fig. 2A. The purity of each sorted population was higher than 98% in all the experiments.

When we analyze the ability of each isolated T cell population to suppress the proliferation of activated T cells, we confirmed the strong suppressive effect of aTregs (Fig. 2B), and the non-suppressive activity of FOXP3+ non-Tregs [5]. In addition to the previously reported capacity to produce substantial amounts of IFN-γ, IL-2, and IL-17 upon stimulation [5], we found that FOXP3+ non-Tregs, but not aTregs or rTregs, produced not only Th1 and Th17 cytokines, but also high levels of Th2 cytokines (IL-5, IL-13), inflammatory cytokines (TNF-α), chemokines (IL-8 and MIP-1β), and growth factors (GM-CSF) (Fig. 2C).

### HIV-1 Infected Patients Show a Decrease Number of Both FOXP3+ Tregs and FOXP3+ non-Tregs

Having characterized the functional profile of the different subsets of FOXP3+CD4+ T cells, we analyzed the status of each cell population in HIV-1-infected patients. The characteristics of the cohort of untreated HIV-1-infected patients are showed in Table 1. Accordingly to widely accepted criteria [18], [19], [20], the HIV-1 cohort was divided into a low CD4 group, with CD4 counts lower than 350 cells/µl (Low, n = 20), and a high CD4 group, with CD4 counts higher than 350 cells/µl (High, n = 35). The observations depicted in Table 2 show that the relative frequency of rTregs and aTregs was similar in HIV-1 patients and healthy donors (HD). In contrast, in comparison with HD, the frequency of FOXP3+ non-Tregs was significantly higher in both groups of HIV-1 patients, reaching a maximum value of 8.5% of the total number of CD4+ T cells in those patients with more advanced stage of HIV-1 infection (those with low CD4 counts).

**Table 2:** Comparison of the frequency and the number of FOXP3+ Tregs, FOXP3+ non-Tregs and FOXP3- naive and memory T cells in healthy donors and HIV-1-infected patients.

	HD	HIV-1		
		All HIV-1	High CD4	Low CD4
	*n* 27	*n* 55	*n* 35	*n* 20
**rTregs (%)** #	1.7±0.1	2±0.2 NS	2.1±0.3 NS	1.7±0.3 NS
**rTregs (cells/µl)** $	17±1	8±1 ***	10±1 **	4±1 ***
**aTregs (%)**	0.6±0.1	0.9±0.13 NS	0.8±0.1 NS	1.2±0.3 NS
**aTregs (cells/µl)**	6±1	3±0.2 ***	3±0.3 **	2±1 ***
**FOXP3+ non-Tregs (%)**	3.3±0.2	6±0.5 ***	4.5±0.3 *	8.5±1.2 ***
**FOXP3+ non-Tregs** **(cells/µl)**	33±2	19±1.2 ***	22±2 ***	14±2 ***
**Naive (%)**	43.3±2.3	31.9±1.5 ***	31.1±1.8 *	26.3±2.2 ***
**Naive (cells/µl)**	418±27	132±13***	170±14 ***	62±9 ***
**Memory (%)**	43.1±2.6	53.9±1.8 **	53.5±2.3 *	54.7±2.8 **
**Memory (cells/µl)**	433±26	212±15 ***	269±16 **	111±11 ***

# Data are expressed as % of all CD4+ T cells.

$ The absolute number for each cell fraction (see Figure 1Ai), is expressed as cells/µl.

* p<0.05,

** p<0.01,

*** p<0.0001.

NS , not significant. Data are analyzed by Mann-Whitney test and Kruskall-Wallis test followed by Dunn multiple comparison post test. Data are presented as the mean ± SEM. Calculated *P* values were determined compared to healthy donors.

Interestingly, the analysis of the absolute number of FOXP3+ T cells revealed that aTregs, rTregs and FOXP3+ non-Tregs were significantly reduced in both HIV-1 groups compared with HD (Table 2).

In agreement with our results, a recent report [21] has shown a decrease number of both rTregs and aTregs in HIV-1-infected patients. However, this previous study did not provide information about the subset of FOXP3+ non-Tregs. In addition, and contrasting with our present results, this study showed that the frequency of aTregs was significantly lower for HIV-1 patients compared with HD [21]. We speculate that the reason underlying this disappointed observation could be related to the different characteristics of the cohort of HIV-1-infected patients used in each case. In our study, the interquartile range (IQR) of CD4+ T cell counts in the cohort analyzed was 273–530 cells/µl, while in the cited study the range of CD4+ T cell counts shows a more heterogenous IQR of 421–1316 cells/µl [21].

Previous works focused on the analysis of regulatory T cells in HIV-1 infection have mostly identified regulatory T cells as FOXP3+CD4+ T cells [10], [11], [12], [13], [14], [16], [22], [23], [24], [25]. Our present results indicate that FOXP3+ non-Tregs do not show a suppressor phenotype but display a very high ability to produce a broad spectrum of cytokines able to induce the differentiation of CD4+ T cells in different effectors profiles as well as to start a variety if innate immune responses. Thus, our results reinforce the notion that in order to evaluate the status of CD4+ suppressor T cells in HIV-1 patients this subset of FOXP3+ non-Tregs should be clearly discriminated from FOXP3+ Tregs. In this regard, it should be emphasized that FOXP3+ non-Tregs represent the large subset of FOXP3+CD4+ T cells in the peripheral blood of either HD or HIV-1-infected patients.

**Figure 3:**
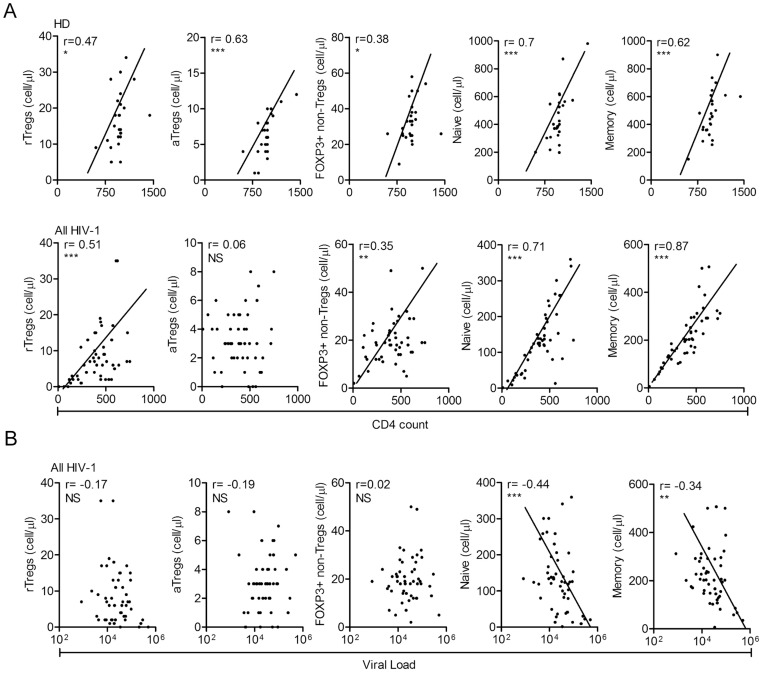
Analysis of the correlation between each T cell subset and the total counts of CD4+ T cells or the virus load. A. A positive correlation between the absolute number of CD4+ T cells and the absolute number of each T cell subset is observed in both HD and HIV-1 patients, except in the aTreg subset in the cohort of HIV-1-infected patients (HD, n = 27; All HIV-1, n = 55; Spearman correlation test). **B.** A negative correlation between FOXP3- naive and memory T cells and viral load that was not observed for the three populations of FOXP3+ T cells (Spearman correlation test). * p<0.05, ** p<0.01, *** p<0.0001, NS = not significant.

We also analyzed the correlation between the absolute counts of all three populations of FOXP3+CD4+ T cells with CD4+ T cell numbers. We found a positive correlation between rTregs and CD4+ T cell counts. However, we did not found any correlation between aTregs and CD4+ T cells (Fig. 3A). This lack of correlation might be reflecting that the mechanisms regulating the homeostasis of aTregs in the peripheral blood of HIV-1 infected patients differ from the other populations of CD4+ T cells. As expected, the number of FOXP3- naive and memory T cells significantly correlated with CD4+ counts. Moreover, when analyzing the correlation between the three populations of FOXP3+CD4+ T cells and the viral load we found no correlation, suggesting that the viral load is not under the regulation of FOXP3+CD4+ T cells. Finally, as widely reported, the number of naive and memory T cells correlate inversely with the viral load, (Fig. 3B).

### aTregs, and FOXP3+ non-Tregs Cells Show a High Susceptibility to HIV-1 Infection

**Figure 4:**
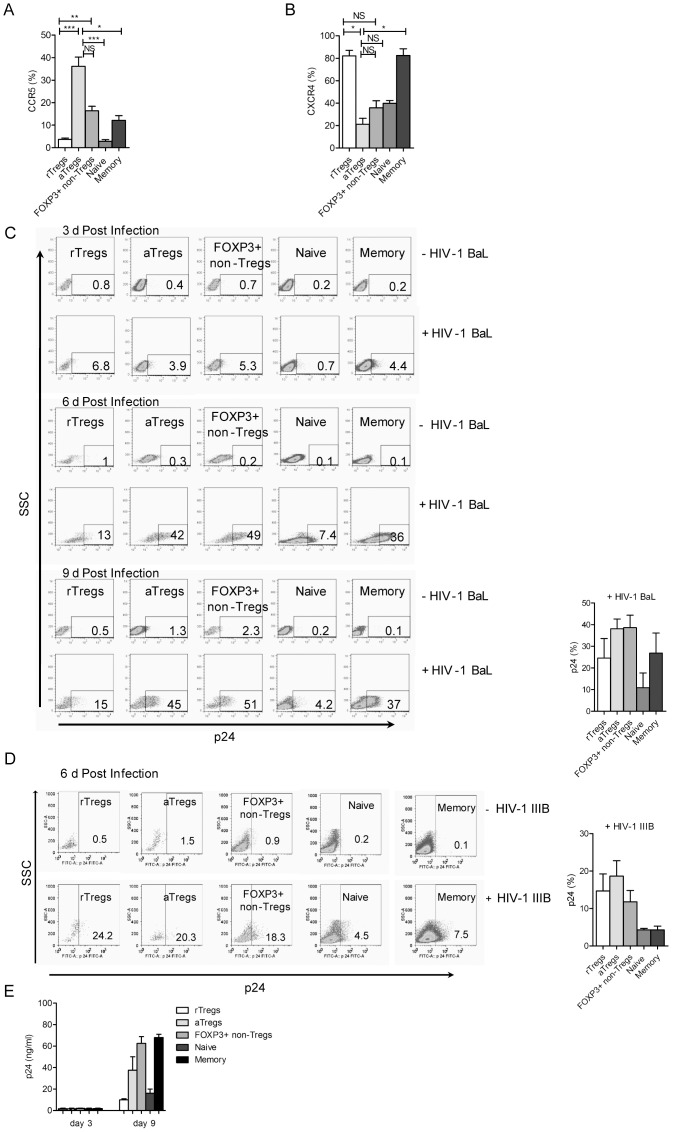
Susceptibility of FOXP3+ T cells to HIV-1 infection. A and B. PBMCs were stained with anti-CD4, anti-CD45RA, anti-FOXP3, and anti-CCR5 or anti-CXCR4 antibodies, and the expression of each HIV-co-receptor was analyzed in rTregs, aTregs, FOXP3+ non-Tregs, naive, and memory T cells. Kruskall-Wallis test followed by Dunn multiple comparison post test (n = 5), * p<0.05, ** p<0.01, *** p<0.0001, NS = not significant. **C and D.** PBMCs were activated with PHA for 2 days and cultured in medium supplemented with IL-2. Cells were then infected with CCR5-using HIV-1 BaL (C) or CXCR4-using HIV-1 IIIB (D). The infection was quantified at day 3, 6 or 9 post-infection by measuring the expression of intracellular p24 by flow cytometry in each of the five populations of CD4+ T cells. Dot-plots (side scatter profile vs p24 antigen) from a representative experiment are shown in the left panels of (C) and (D). The percentages of positive cells for p24 expression are shown at the bottom right boxes. The mean ± SEM of 6 experiments are shown in the right panels of (C) and (D). **E.** The five populations of sorted CD4+ T cells were activated with PHA for 2 days and then infected with HIV-1 BaL. The levels of p24 antigen in cell supernatants were measured at 3 and 9 days post-infection by ELISA. Results are the mean ± SEM of 5 experiments.

No previous studies have analyzed the susceptibility of FOXP3+ Tregs and FOXP3+ non-Tregs to HIV-1 infection. In a first set of experiments we analyzed the expression of the co-receptors CCR5 and CXCR4 in each cell population by staining PBMCs with the combination of anti-CD4, anti-CD45RA, anti-FOXP3, and anti-CCR5 or anti-CXCR4 antibodies. The highest expression of CCR5 was detected on aTregs (36±4%), followed by FOXP3+ non-Tregs (16.4±2%) and rTregs (3.7±0.6%). The expression of CCR5 in memory and naive T cells was 12.2±2% and 2.9±0.8%, respectively (Fig. 4A). On the other hand, the expression of CXCR4 was higher in rTregs (82.1±4.9%) and memory T cells (82.5±6.1%), compared with the other cell populations.

In a second set of experiments we infected activated-PBMCs by HIV-1. The infection of the different populations of CD4+ T cells was analyzed by measuring intracellular p24 antigen by flow cytometry at 3, 6, and 9 days post-infection. Using HIV-1 BaL virus we found that aTregs and FOXP3+ non-Tregs were highly permissively to HIV-1 infection (p<0.003). A representative FACS profile is depicted in Fig. 4C (left panel) and the mean percentages of infected cells observed in 6 different experiments at 9 day post-infection is showed in Fig. 4C (right panel). On the other hand, using the HIV-1 IIIB virus we found that all FOXP3+ T cells were similarly permissive to HIV-1 infection, being the percentages of infection significantly higher (p<0.002) for the three FOXP3+ T cell subsets compared with conventional T cells, (Fig. 4D, right and left panels).

To further analyze the susceptibility of each population of FOXP3+ CD4+ T cells to HIV-1 infection, we next performed additional infection assays using sorted populations of CD4+ T cells, obtained as described above. Each of the five populations of sorted CD4+ T cells were activated with PHA for 2 days and then infected with HIV-1 BaL. Cell infection was evaluated at 3 and 9 days post-infection by measuring the levels of p24 antigen in cell supernatants by ELISA. Consistent with the data obtained with non-sorted PBMCs showed in Fig. 4C, the results depicted in Fig. 4E shows that both aTregs and FOXP3+ non-Tregs are highly permissive to HIV-1 infection.

**Figure 5:**
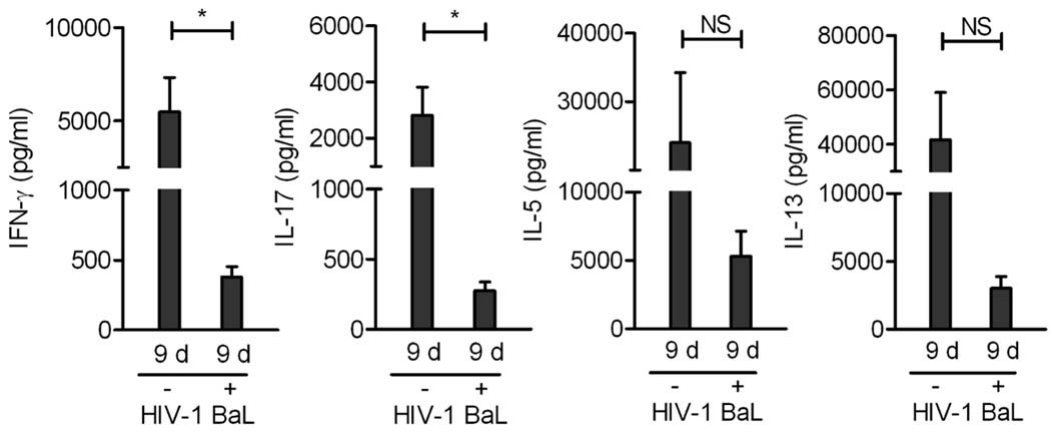
Effect of HIV-1 infection on the production of Th1, Th17, and Th2 cytokines by FOXP3+ non-Tregs. The cell sorting strategy to obtain FOXP3+ non-Tregs from HD was described in Figure 2. Sorted FOXP3+ non-Tregs from HD were activated with PHA for 2 days and then infected with CCR5-using HIV-1 BaL. At day 9, cells were re-stimulated with PMA/iono for 5 h, and the production of cytokines was evaluated in cell supernatants by using a Bioplex system. Results are the mean ± SEM of 5 independent experiments. * p<0.05, NS = not significant.

Considering the high ability of FOXP3+ non-Tregs to produce Th1, Th17, and Th2 cytokines, we next analyzed whether infection was able to modulate the profile of cytokines produced by sorted FOXP3+ non-Tregs. Figure 5 shows that infection by HIV-1 BaL down-regulated the production of all the cytokines assessed; IFN-γ, IL-17, IL-5, and IL-13. However, only the reduction of IFN-γ and IL-17 reached statistical significance. More importantly, after HIV-1 infection FOXP3+ non-Tregs retained their ability to produce substantial amounts of IL-5 and IL-13, but not IFN-γ and IL-17, suggesting that under the influence of HIV-1 infection FOXP3+ non-Tregs might contribute to the promotion of Th2 responses.

In summary, our study shed light about some important properties of FOXP3+ non-Tregs. We demonstrate that they are able to produce a variety of cytokines, including Th1, Th2, Th17, inflammatory cytokines, chemokines and growth factors. The wide spectrum of cytokines secreted by FOXP3+ non-Tregs may indicate that these cells represent an early stage of differentiation after activation of naive T cells [26], which precede the more differentiated subsets of effectors T cells, like Th1, Th2 or Th17 cells. We also show that the frequency of this cellular subset markedly increases in the group of HIV-1-infected patients with low values of CD4 counts. FOXP3+ non-Tregs showed a high susceptibility to the infection by both R5- and X4-tropic HIV-1 and, as a consequence of HIV-1 infection, they preserve a pattern of cytokine production able to promote Th2 responses. It is well known that HIV-1 infection is associated with a dysregulation of the cytokine network that contributes to AIDS. The increased frequency of Th2 cells in patients that failed to achieve a sustained control of virus, suggests that polarization toward a Th2-mediated response plays a critical role in HIV-1 progression [27], [28], [29]. Our present results suggest that FOXP3+ non-Tregs might contribute to the polarization of CD4+ T cells into a Th2 profile, predictive of a poor outcome of HIV-1-infected patients.

## References

[1] Fontenot JD, Gavin MA, Rudensky AY (2003) Foxp3 programs the development and function of CD4+CD25+ regulatory T cells. Nat Immunol 4: 330–336. 12612578 10.1038/ni904

[2] Roncador G, Brown PJ, Maestre L, Hue S, Martinez-Torrecuadrada JL, et al (2005) Analysis of FOXP3 protein expression in human CD4+CD25+ regulatory T cells at the single-cell level. Eur J Immunol 35: 1681–1691. 15902688 10.1002/eji.200526189

[3] Liu W, Putnam AL, Xu-Yu Z, Szot GL, Lee MR, et al (2006) CD127 expression inversely correlates with FoxP3 and suppressive function of human CD4+ T reg cells. J Exp Med 203: 1701–1711. 16818678 10.1084/jem.20060772 PMC2118339

[4] Seddiki N, Santner-Nanan B, Martinson J, Zaunders J, Sasson S, et al (2006) Expression of interleukin (IL)-2 and IL-7 receptors discriminates between human regulatory and activated T cells. J Exp Med 203: 1693–1700. 16818676 10.1084/jem.20060468 PMC2118333

[5] Miyara M, Yoshioka Y, Kitoh A, Shima T, Wing K, et al (2009) Functional delineation and differentiation dynamics of human CD4+ T cells expressing the FoxP3 transcription factor. Immunity 30: 899–911. 19464196 10.1016/j.immuni.2009.03.019

[6] Douek DC, Picker LJ, Koup RA (2003) T cell dynamics in HIV-1 infection. Annu Rev Immunol 21: 265–304. 12524385 10.1146/annurev.immunol.21.120601.141053

[7] Mattapallil JJ, Douek DC, Hill B, Nishimura Y, Martin M, et al (2005) Massive infection and loss of memory CD4+ T cells in multiple tissues during acute SIV infection. Nature 434: 1093–1097. 15793563 10.1038/nature03501

[8] Belkaid Y, Tarbell K (2009) Regulatory T cells in the control of host-microorganism interactions (*). Annu Rev Immunol 27: 551–589. 19302048 10.1146/annurev.immunol.021908.132723

[9] Sakaguchi S (2005) Naturally arising Foxp3-expressing CD25+CD4+ regulatory T cells in immunological tolerance to self and non-self. Nat Immunol 6: 345–352. 15785760 10.1038/ni1178

[10] Epple HJ, Loddenkemper C, Kunkel D, Troger H, Maul J, et al (2006) Mucosal but not peripheral FOXP3+ regulatory T cells are highly increased in untreated HIV infection and normalize after suppressive HAART. Blood 108: 3072–3078. 16728694 10.1182/blood-2006-04-016923

[11] Eggena MP, Barugahare B, Jones N, Okello M, Mutalya S, et al (2005) Depletion of regulatory T cells in HIV infection is associated with immune activation. J Immunol 174: 4407–4414. 15778406 10.4049/jimmunol.174.7.4407

[12] Tenorio AR, Martinson J, Pollard D, Baum L, Landay A (2008) The relationship of T-regulatory cell subsets to disease stage, immune activation, and pathogen-specific immunity in HIV infection. J Acquir Immune Defic Syndr 48: 577–580. 18645514 10.1097/QAI.0b013e31817bbea5

[13] Schulze Zur Wiesch J, Thomssen A, Hartjen P, Toth I, Lehmann C, et al (2011) Comprehensive analysis of frequency and phenotype of T regulatory cells in HIV infection: CD39 expression of FoxP3+ T regulatory cells correlates with progressive disease. J Virol 85: 1287–1297. 21047964 10.1128/JVI.01758-10 PMC3020516

[14] Lim A, Tan D, Price P, Kamarulzaman A, Tan HY, et al (2007) Proportions of circulating T cells with a regulatory cell phenotype increase with HIV-associated immune activation and remain high on antiretroviral therapy. AIDS 21: 1525–1534. 17630546 10.1097/QAD.0b013e32825eab8b

[15] Fazekas de St Groth B, Landay AL (2008) Regulatory T cells in HIV infection: pathogenic or protective participants in the immune response? AIDS 22: 671–683. 18356596 10.1097/QAD.0b013e3282f466da

[16] Bi X, Suzuki Y, Gatanaga H, Oka S (2009) High frequency and proliferation of CD4+ FOXP3+ Treg in HIV-1-infected patients with low CD4 counts. Eur J Immunol 39: 301–309. 19089812 10.1002/eji.200838667

[17] Arruvito L, Sanz M, Banham AH, Fainboim L (2007) Expansion of CD4+CD25+and FOXP3+ regulatory T cells during the follicular phase of the menstrual cycle: implications for human reproduction. J Immunol 178: 2572–2578. 17277167 10.4049/jimmunol.178.4.2572

[18] Severe P, Juste MA, Ambroise A, Eliacin L, Marchand C, et al (2010) Early versus standard antiretroviral therapy for HIV-infected adults in Haiti. N Engl J Med 363: 257–265. 20647201 10.1056/NEJMoa0910370 PMC3676927

[19] Hammer SM, Eron JJ Jr, Reiss P (2008) Schooley RT, Thompson MA, et al (2008) Antiretroviral treatment of adult HIV infection: 2008 recommendations of the International AIDS Society-USA panel. JAMA 300: 555–570. 18677028 10.1001/jama.300.5.555

[20] Lundgren JD, Battegay M, Behrens G, De Wit S, Guaraldi G, et al (2008) European AIDS Clinical Society (EACS) guidelines on the prevention and management of metabolic diseases in HIV. HIV Med 9: 72–81. 18257770 10.1111/j.1468-1293.2007.00534.x

[21] Simonetta F, Lecuroux C, Girault I, Goujard C, Sinet M, et al (2012) Early and long-lasting alteration of effector CD45RA(-)Foxp3(high) regulatory T-cell homeostasis during HIV infection. J Infect Dis 205: 1510–1519. 22457280 10.1093/infdis/jis235 PMC3989210

[22] Apoil PA, Puissant B, Roubinet F, Abbal M, Massip P, et al (2005) FOXP3 mRNA levels are decreased in peripheral blood CD4+ lymphocytes from HIV-positive patients. J Acquir Immune Defic Syndr 39: 381–385. 16010156 10.1097/01.qai.0000169662.30783.2d

[23] Andersson J, Boasso A, Nilsson J, Zhang R, Shire NJ, et al (2005) The prevalence of regulatory T cells in lymphoid tissue is correlated with viral load in HIV-infected patients. J Immunol 174: 3143–3147. 15749840 10.4049/jimmunol.174.6.3143

[24] Baker CA, Clark R, Ventura F, Jones NG, Guzman D, et al (2007) Peripheral CD4 loss of regulatory T cells is associated with persistent viraemia in chronic HIV infection. Clin Exp Immunol 147: 533–539. 17302904 10.1111/j.1365-2249.2006.03319.x PMC1810503

[25] Hunt PW, Landay AL, Sinclair E, Martinson JA, Hatano H, et al (2011) A low T regulatory cell response may contribute to both viral control and generalized immune activation in HIV controllers. PLoS One 6: e15924. 21305005 10.1371/journal.pone.0015924 PMC3031543

[26] Di Mitri D, Azevedo RI, Henson SM, Libri V, Riddell NE, et al (2011) Reversible senescence in human CD4+CD45RA+CD27- memory T cells. J Immunol 187: 2093–2100. 21788446 10.4049/jimmunol.1100978

[27] Imami N, Pires A, Hardy G, Wilson J, Gazzard B, et al (2002) A balanced type 1/type 2 response is associated with long-term nonprogressive human immunodeficiency virus type 1 infection. J Virol 76: 9011–9023. 12186885 10.1128/JVI.76.18.9011-9023.2002 PMC136425

[28] Sousa AE, Chaves AF, Doroana M, Antunes F, Victorino RM (1999) Kinetics of the changes of lymphocyte subsets defined by cytokine production at single cell level during highly active antiretroviral therapy for HIV-1 infection. J Immunol 162: 3718–3726. 10092835

[29] Tsunemi S, Iwasaki T, Imado T, Higasa S, Kakishita E, et al (2005) Relationship of CD4+CD25+ regulatory T cells to immune status in HIV-infected patients. AIDS 19: 879–886. 15905668 10.1097/01.aids.0000171401.23243.56

